# Risk stratification and mortality prediction in octo- and nonagenarians with peripheral artery disease: a retrospective analysis

**DOI:** 10.1186/s12872-021-02177-1

**Published:** 2021-08-02

**Authors:** Christos Rammos, Aristotelis Kontogiannis, Amir A. Mahabadi, Martin Steinmetz, Daniel Messiha, Julia Lortz, Tienush Rassaf

**Affiliations:** Department of Cardiology and Vascular Medicine, West German Heart and Vascular Center Essen, University Hospital Essen, University of Duisburg-Essen, Hufelandstraße 55, 45147 Essen, Germany

**Keywords:** Aging, Peripheral interventions, Endovascular treatment, Mortality

## Abstract

**Objectives:**

Among changes in demographics, aging is the most relevant cardiovascular risk factor. The prevalence of peripheral artery disease (PAD) is high in elderly patients and is associated with a worse prognosis. Despite optimal treatments, mortality in the high-risk population of octo- and nonagenarians with PAD remains excessive, and predictive factors need to be identified. The objective of this study was to investigate predictors of mortality in octo- and nonagenarians with PAD.

**Methods:**

Cases of treated octo- and nonagenarians, including the clinical characteristics and markers of myocardial injury and heart failure, were studied retrospectively with respect to all-cause mortality. Hazard ratios [HR] were calculated and survival was analyzed by Kaplan-Meyer curves and receiver operating characteristic curved were assessed for troponin-ultra and N-terminal pro-brain natriuretic peptide (NT-proBNP) levels and chronic limb-threatening ischemia (CLTI).

**Results:**

A total of 123 octo- and nonagenarians admitted for PAD were eligible. The troponin level was the major predictor of all-cause mortality (HR: 4.6, 95% confidence interval [CI]: 1.4–15.3), followed by the NT-proBNP level (HR: 3.9, 95% CI 1.8–8.8) and CLTI (HR: 3.1, 95% CI 1.6–5.9). Multivariate regression revealed that each increment of 1 standard deviation in log troponin and log NT-proBNP was associated with a 2.7-fold (95% CI 1.8–4.1) and a 1.9-fold (95% CI 1.2–2.9) increased risk of all-cause death. Receiver operating characteristic curve analysis using a combination of all predictors yielded an improved area under the curve of 0.888. In a control group of an equal number of younger individuals, only NT-proBNP (HR: 4.2, 95% CI 1.2–14.1) and CLTI (HR: 6.1, 95% CI 1.6–23.4) were predictive of mortality.

**Conclusion:**

Our study demonstrates that cardiovascular biomarkers and CLTI are the primary predictors of increased mortality in elderly PAD patients. Further risk stratification through biomarkers in this high-risk population of octo- and nonagenarians with PAD is necessary.

**Supplementary Information:**

The online version contains supplementary material available at 10.1186/s12872-021-02177-1.

## Introduction

Aging is inevitable. The global share of people aged 65 years or over is expected to double from 12% in 2010 to 22% by 2040 [1, 2]. Aging represents a key cardiovascular risk factor and is associated with alterations of the cardiovascular system [3, 4]. Atherosclerotic cardiovascular disease (CVD) is a leading cause of death, and more than two-thirds of CVD-related deaths occur in the geriatric population [5].

Atherosclerotic CVD is characterized by atheromatous plaques that lead to blood flow restrictions and eventually tissue ischemia. Peripheral artery disease (PAD) is a major noncoronary manifestation of CVD. The 2016 European guidelines on CVD prevention in clinical practice place patients with PAD in the very high-risk category [6].

Despite optimal treatment, the elderly population remains at an increased risk for all-cause mortality. In addition to pharmacotherapy, treatment of symptomatic PAD through minimally invasive endovascular techniques has gained widespread acceptance and is the primary revascularization strategy for reconstituting perfusion [7]. Moreover, modern treatments for CVD have helped extend life expectancy [8]. Both facts have led to an increased number of elderly patients referred for endovascular treatment due to atherosclerotic CVD. Elderly patients now constitute more than one in five patients treated with percutaneous interventions in hospitals worldwide [9]. Furthermore, octogenarians seem to have more complex lesions than their younger counterparts and they are undergoing more complex interventional procedures than before [10].

Traditional cardiovascular risk factors have been implicated in prevalent and incident PAD, and comorbidities are common in affected patients [11]. Accurate risk stratification and mortality prediction are essential, as these aid the clinical decision-making process, help physicians estimate the need for resources and facilitate proper patient counseling. The aim of the present study was thus to investigate predictors of mortality in octo- and nonagenarians with PAD.

## Methods

We retrospectively reviewed patient files and identified a total of 1808 patients who were admitted for PAD treatment between January 2016 and December 2017 at the Department of Vascular Medicine of a university tertiary referral center. Patients with PAD undergoing nonemergent and emergent vascular interventions were eligible if they suffered from moderate to severe intermittent claudication or chronic limb-threatening ischemia (CLTI) with ischemic rest pain or minor or major ulcers (Rutherford grade 2–6). Patients primarily admitted for vascular surgery were excluded.

Endovascular interventional procedures were conducted at a single tertiary referral center and were performed by experienced interventionalists. The use of commercially available drug-coated balloons, self- and balloon-expanding stents and devices for mechanical debulking and atherectomy were allowed at the physician’s discretion in order to achieve the best clinical results.

Standard techniques for the determination of peripheral perfusion were applied according to the current guidelines, as previously described [12–14]. For the ankle-brachial index (ABI), the systolic blood pressure was assessed using a pneumatic cuff and Doppler measurement at the brachial, dorsalis and posterior tibial arteries (left and right). The ABI was determined by calculating the ratio of the highest systolic pressure at the brachial artery to the highest systolic pressure at the ankle. The measurements were performed at standardized room temperature after 15 min of acclimatization.

### Clinical and laboratory measurements

Data were collected by interview, physical examination and laboratory analysis. Data included demographic data, past medical history, drug consumption and smoking behavior. Blood pressure and anthropometric measurements were collected during the physical examination. Blood was drawn per routine clinical practice, and the local University Hospital Institute of Clinical Chemistry and Laboratory Diagnostics performed all analyses.

### Statistics

Continuous variable are expressed as mean ± SD, and categorical variables are expressed as percentages. Comparisons between continuous data were made by Student’s t-test. Survival analysis was conducted using Kaplan–Meier curves. Hazard ratios (HRs) were calculated using the log-rank (Mantel-Cox) test. For the N-terminal pro-brain natriuretic peptide (NT-proBNP) level, we chose to use age-dependent cutoff values, as recently suggested in the literature [15]. We further examined the risk associated with the continuous values of troponin-ultra and NT-proBNP after log transformation to normalize the distribution and determine the risk per standard deviation (SD) increase in the log troponin and log NT-proBNP levels. The sensitivity and specificity of expected versus observed mortality are summarized by receiver operator characteristic (ROC) curves and the area under the ROC curve (AUROC). To further evaluate the magnitude of risk factors on mortality we further performed a stepwise multivariate regression. P values of less than 0.05 were regarded as statistically significant. All statistical tests were conducted using SPSS 21.0 (IBM) and Prism 8.0 (GraphPad).

## Results

Overall, the files of 1808 patients admitted to the department of vascular medicine for the endovascular treatment of symptomatic PAD were screened. Of those, 123 patients aged 80 years and older with PAD as the primary diagnosis at the time of admission and with adequate follow-up data were eligible and compared to consecutively admitted nonmatched patients under age 80 (n = 123) referred for subsequent peripheral intervention in 2016.

**Fig. 1:**
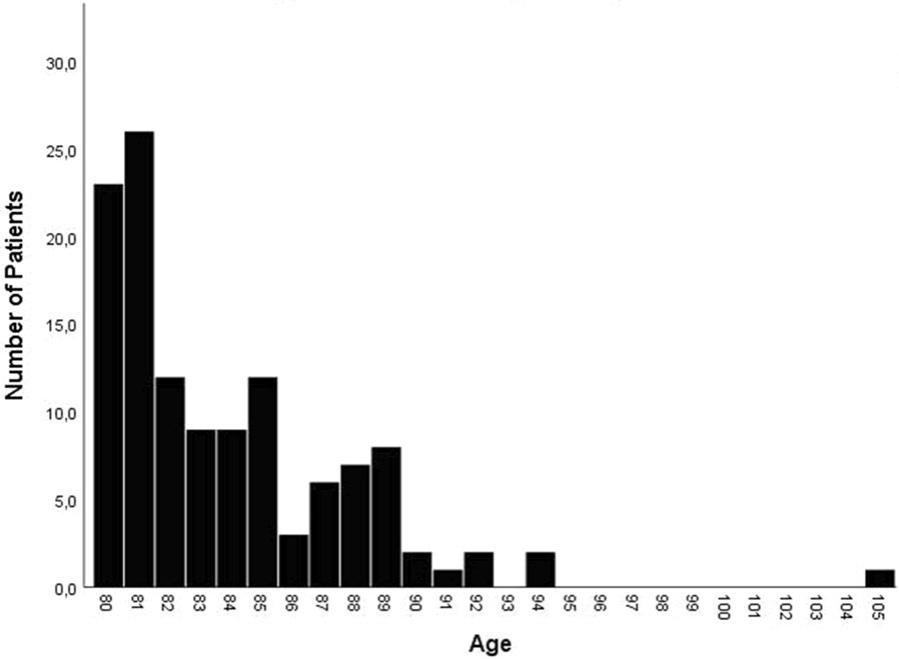
Age-related distribution of octo- and nonagenarians

All patients underwent vascular interventions due to symptomatic PAD. Octo- and nonagenarian patients were 84 ± 4 years old, with a body mass index of 26 ± 5 kg/m^2^; 53% were women. Fifty-eight percent of the patients aged 80 years and older were admitted with CLTI and minor or major tissue loss, while only 42% had intermittent claudication. Younger patients were predominantly admitted due to intermittent claudication (76%), and 24% were admitted due to CLTI (*p* = 0.0001). Octo- and nonagenarians were less frequently diagnosed with coronary artery disease than younger patients (47% vs. 61%, respectively; *p* = 0.029). Further baseline characteristics are shown in Table 1, and the age-related distribution is depicted in Fig. 1.

**Table 1:** Demographic data

	≥ 80 years old (n = 123)	< 80 years old (n = 123)	*P* value
Age, years	84 ± 4	66 ± 9	0.0001
Sex (female)	65 (53%)	35 (28%)	0.0001
Body mass index, kg/m^2^	26 ± 5	27 ± 5	0.012
Hypertension	110 (89%)	112 (91%)	0.83
Diabetes mellitus	38 (31%)	51 (41%)	0.196
Obesity	16 (13%)	24 (20%)	0.011
Current smoking	12 (10%)	45 (37%)	0.0001
ABI target limb	0.7 ± 0.4	0.67 ± 0.3	0.372
Coronary artery disease	58 (47%)	76 (61%)	0.029
History of MI	14 (11%)	18 (15%)	0.570
Prior CABG	17 (14%)	18 (15%)	0.235
History of stroke	11 (9%)	18 (15%)	0.172
Atrial fibrillation	40 (32%)	12 (10%)	0.0001
COPD	14 (11%)	28 (23%)	0.027
Kidney disease G3a and over	74 (60%)	23 (19%)	0.0001
Regular hemodialysis	5 (4%)	2 (2%)	0.749
PAD stage (Rutherford)			
1–3	52 (42%)	94 (76%)	0.0001
4–6	71 (58%)	29 (24%)	
Prior PTA in the last 3 months	4 (3%)	24 (20%)	0.0001
Prior peripheral bypass	17 (14%)	18 (15%)	0.235
Antiplatelet therapy	82 (67%)	110 (90%)	0.0001
Statin therapy	79 (64%)	95 (77%)	0.035

Clinical characteristics and medications of patients with PAD

Data are given as *n* ± SD (%), unless otherwise stated

PAD, peripheral artery disease; ABI, ankle-brachial index; MI, myocardial infarction; CABG, coronary artery bypass grafting; PTA, percutaneous transluminal angioplasty; BMI, body mass index; MI, myocardial infarction

Octo- and nonagenarians had a mean of troponin-ultra levels of 528 ng/l ± 4500 ng/l and NT-proBNP levels of 3120 pg/ml ± 6158 pg/ml. Notably, these were not different from those observed in the younger PAD patients (troponin-ultra: 32 ng/l ± 250 ng/l, *p* = 0.25; NT-proBNP: 4255 pg/ml ± 29,427 pg/ml, *p* = 0.79). The low-density lipoprotein (LDL) level was also similar in octo- and nonagenarians (111 ± 44 mg/dl) and their younger counterparts (107 ± 37 mg/dl, *p* = 0.53). Alarmingly, a lower proportion of octo- and nonagenarians than younger patients were receiving antiplatelet therapy (67% vs 90%, *p* = 0.0001). This undertreatment of octo- and nonagenarians was also observed regarding statin therapy (64% in octogenarians and 77% in younger counterparts, *p* = 0.035; Table 1). Further data regarding baseline laboratory characteristics, medications, and procedural characteristics are shown in Additional file 1: Table S1, Table 2 and Additional file 1: Table S2, respectively.

**Table 2:** Baseline medications

	≥ 80 years old (n = 123)	< 80 years old (n = 123)	
Antiplatelet therapy	82 (67%)	110 (90%)	0.0001
VKA	15 (12%)	10 (8%)	0.0001
DOACs	28 (23%)	2 (2%)	0.0001
Statin therapy	79 (64%)	95 (77%)	0.035
ACE- inhibitor	57 (46%)	64 (52%)	0.444
AT-II-receptor antagonist	31 (25%)	34 (28%)	0.773
Beta-blockers	73 (60%)	80 (65%)	0.430
Calcium channel blockers	47 (38%)	47 (38%)	1
Aldosterone antagonists	18 (15%)	13 (11%)	0.443
Thiazides	35 (28%)	30 (24%)	0.563
Loop diuretics	55 (45%)	35 (28%)	0.008

VKA, Vitamin K antagonists; DOACs, Directs oral anticoagulants; ACE, angiotensin converting enzyme

Retrospective analysis yielded a mean follow-up of 12.5 months (range: 0.1–24 months). During this period, 35 (28%) participants in the octogenarian group and 13 (10%) of their younger counterparts died.

**Fig. 2:**
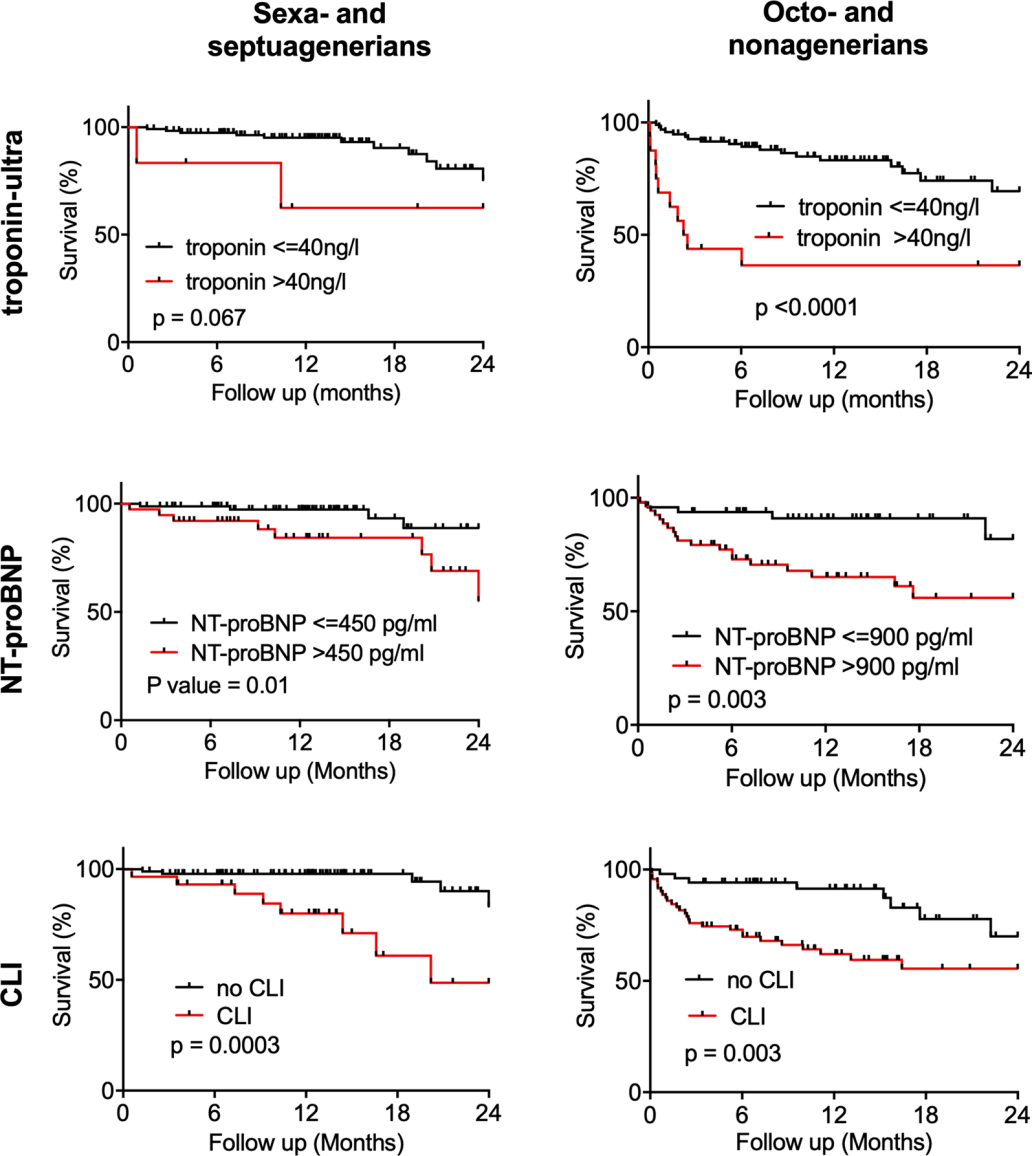
Predictors of mortality in octo- and nonagenarians and younger PAD patients. Survival of PAD patients and predictors of mortality in octo- and nonagenarians and younger patients patients stratified according to troponin-ultra values, age-adjusted NT-proBNP values and CLTI

Survival was analyzed according to the troponin-ultra level, NT-proBNP level and CLTI. For all predictors investigated, a significant relation was revealed in the octo- and nonagenarian population, with the strongest predictor being a troponin-ultra level above the 99^th^ percentile of 40 ng/l (HR: 4.6, 95% CI 1.4–15.3, *p* < 0.0001), followed by an NT-proBNP level of above 900 pg/ml (HR: 3.9, 95% CI 1.8–8.8, *p* = 0.003) and CLTI (HR: 3.1, 95% CI 1.6–5.9, *p* = 0.004; Fig. 2). In contrast, in younger patients, only an age-adjusted NT-proBNP level above 450 pg/ml (HR: 4.2, 95% CI 1.2–14.1, *p* = 0.01) and CLTI (HR: 6.1, 95% CI 1.6–23.4, *p* = 0.0003), but not a troponin-ultra level above 40 pg/l (*p* = 0.07), were significantly associated with increased mortality (Fig. 2).

**Fig. 3:**
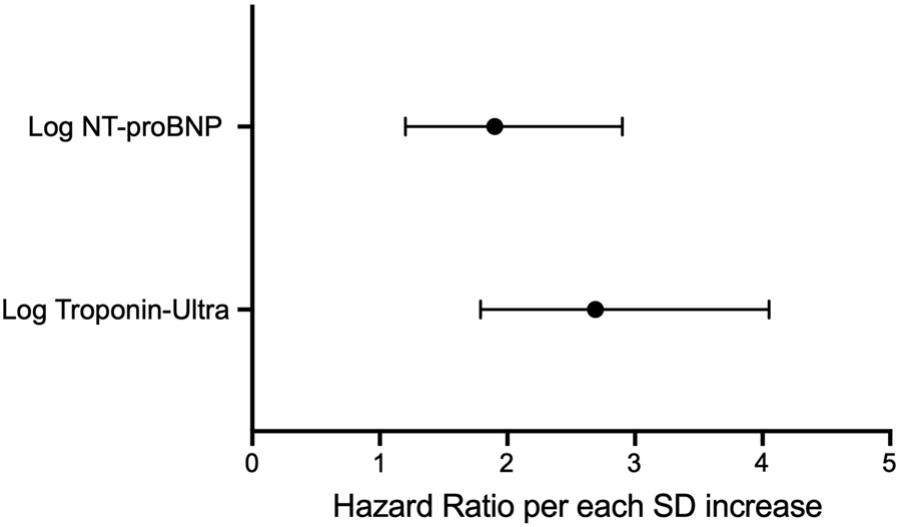
Continuous biomarker values predict mortality in octo- and nonagenarians. Hazard ratio (and 95% confidence intervals) per each SD increase in log troponin-ultra and log NT-proBNP for death from any cause in octo- and nonagenarians

We then analyzed the predictive capabilities of the identified risk factors through Cox regression models. To do this, we additionally evaluated the continuous troponin-ultra and NT-proBNP values and found that each SD increase in log troponin-ultra and log NT-proBNP was associated with an increased HR. Among biomarkers, the log troponin-ultra level was identified as the strongest predictor (HR: 2.7, 95% CI 1.8–4.1), followed by the log NT-proBNP level (HR: 1.9, 95% CI 1.2–2.9), with CLTI as a confounding factor (Fig. 3).

**Fig. 4:**
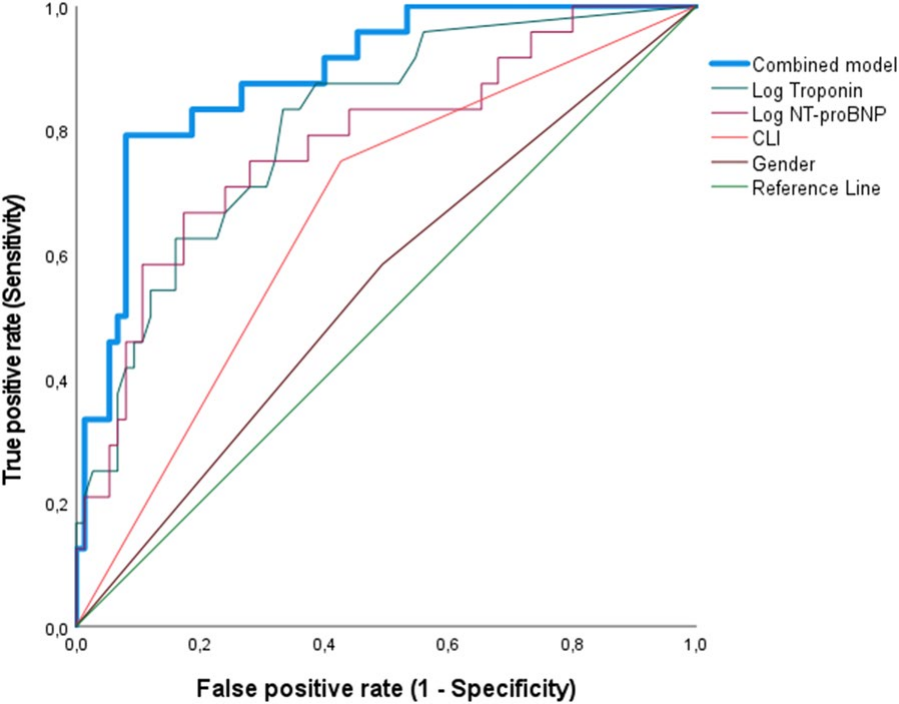
ROC curve analysis for predictability of all-cause mortality in octo- and nonagenarians. ROC curve analysis showing the prognostic value of sex, CLTI, log NT-proBNP, and log troponin-ultra as single factors and a combination model of all factors (AUROC: 0.888)

The AUROC was further evaluated for octo- and nonagenarians and younger counterparts to determine the additional prognostic value of troponin-ultra, NT-proBNP, CLTI, and sex. The ROC curves for octo- and nonagenarians are shown in Fig. 4, and those for younger patients are shown in Additional file 1: Figure S1. Patients aged 80 years and older had an AUROC of 0.806 for log troponin-ultra (*p* = 0.0001 for asymptotic significance, 95% CI 0.7–0.9), 0.779 for log NT-proBNP (*p* = 0.0001 for asymptotic significance, 95% CI 0.67–0.89), 0.662 for CLTI (*p* = 0.003 for asymptotic significance, 95% CI 0.56–0.77) and 0.545 for the female sex (*p* = 0.508, 95% CI 0.413–0.677; Fig. 4).

The characteristics of the combination model were analyzed through the sequential addition of each variable to determine the model's cumulative predictive abilities. The addition of the troponin-ultra level to the NT-proBNP level and clinical stage was significantly more predictive of adverse cardiovascular outcomes than only one factor (AUROC: 0.888 for the combined model of troponin-ultra, NT-proBNP, Rutherford grade and sex, *p* = 0.0001 for asymptotic significance, CI: 0.82–0.96).

ROC curve analysis for younger patients yielded significant AUROC values only for log troponin-ultra (AUROC: 0.688, *p* = 0.041 for asymptotic significance, 95% CI 0.51–0.87), while log NT-proBNP (AUROC: 0.670, *p* = 0.06 for asymptotic significance, 95% CI 0.44–0.89), CLTI (AUROC: 0.6769, *p* = 0.054 for asymptotic significance, 95% CI 0.49–0.86), sex (AUROC: 0.556, *p* = 0.54, 95% CI 0.39–0.73) and the combined model (AUROC: 0.746, *p* = 0.07 for asymptotic significance, 95% CI 0.56–0.77) were not predictive.

To further determine the risk factor with the greatest magnitude of effect on mortality in patients with PAD we performed a stepwise multivariate regression. Analyses were performed separately on the elderly patients and the group of their younger counterparts (< 80 years old). Regarding elderly patients, troponin-ultra, NT-proBNP and CLTI showed a statistically significant influence on mortality (adjusted R square 0.308, sig. F change 0.034). Troponin-ultra showed the greatest magnitude of influence (R square change 0.239, sig. F change < 0.001) followed by NT-proBNP and CLTI (Table 3). Gender, Diabetes, Smoking Status and mild to severe renal failure were excluded from the model as they were non-significant independent variables (univariate analysis Additional file 1: Table S3).

**Table 3:** Multivariate analysis of mortality of elderly patients with PAD

Model	Adjusted R^2^	R^2^ change	Sig. F Change
Troponin I Ultra	0.231	0.239	< 0.001
Troponin I Ultra and NTproBNP blood levels	0.281	0.058	0.006
Troponin I Ultra, NTproBNP and Rutherford stage	0.308	0.033	0.034

In the regression analysis of younger patients, troponin-ultra and CLTI displayed a significant influence on mortality (adjusted R square 0.102, sig. F change 0.032). Troponin-ultra showed the greatest magnitude (R square change 0.081, sig. F change 0.002) followed by CLTI (Table 4). NT-proBNP, gender, smoking status, diabetes and renal failure were excluded from the model as non-significant independent variables.

**Table 4:** Multivariate analysis of mortality of younger patients with PAD

Model	Adjusted R^2^	R^2^ change	Sig. F Change
Troponin I Ultra	0.073	0.081	0.002
Troponin I Ultra and Rutherford stage	0.102	0.036	0.032

## Discussion

Age-related changes in the cardiovascular system are the basis of the increased risk for CVD and lead to clinical vascular and cardiac diseases [16, 17]. Among CVD entities, a major form of atherosclerosis is PAD. PAD itself is a highly prevalent atherosclerotic syndrome and is associated with significant morbidity and mortality. This increased mortality is even higher in elderly patients [18]. Both age and PAD potentiate increased risk; thus, elderly persons with PAD face an increased risk for cardiovascular-related and all-cause mortality [19]. This group of patients, however, is increasingly treated in hospitals worldwide due to demographic changes and improved life expectancy.

Novel risk stratifications have been implemented for many CVDs and procedures, resulting in a treatment paradigm shift toward less invasive or noninvasive strategies [20–22]. Considering peripheral interventions, on the one hand, elderly patients are characterized by more cardiovascular risk factors and a greater burden of atherosclerotic disease than their younger counterparts and older individuals with symptomatic PAD would therefore benefit more from revascularization and reperfusion strategies. On the other hand, they are also more prone to procedural complications due to age-related frailty and comorbidities. Elderly patients treated by coronary percutaneous interventions have recently been shown to have a greater risk for stroke and even a reduced survival rate [23]. Moreover, patients aged 80 years and older undergoing peripheral vascular interventions have a dramatically increased risk for major complications and are more likely to experience vascular access complications [24, 25]. Based on this knowledge, we aimed to investigate a risk prediction model for a hard clinical end point, namely, all-cause mortality, which has not been evaluated before in this at-risk elderly population.

We chose to analyze the predictive capabilities of troponin and NT-proBNP regarding all-cause mortality, as both have previously been shown to be associated with incident PAD and particularly with incident CLTI [26]. While both markers are known to be associated with atherosclerotic diseases and subclinical peripheral atherosclerosis, a potential link has emerged through the concept of impaired ventricular-arterial (VA) coupling [26]. Aortic and arterial functions are known to impact cardiac functions in hypertension, heart failure and cardiac diseases through altered wave reflections and central hemodynamics with an impact on the cardiac afterload [27, 28]. This concept could also be relevant to PAD, but detailed data are lacking. Nonetheless, while we cannot specify the underlying mechanism, it is tempting to speculate that a diseased arterial tree impacts the cardiac system through disturbed VA coupling. Clearly, more investigations need to be conducted to elucidate these potentially highly relevant mechanisms.

In our effort to establish a predictive model of mortality risk, we identified three independent predictive variables in our cohort: troponin, NT-proBNP and CLTI. Our data are supported by studies showing increased mortality in PAD patients aged 75 years and older with increased BNP values [29]. Notably, in other studies with longer follow-up periods, the 5-year mortality rate was 38% for elderly nondiabetic patients and 52% in diabetic patients aged 75 years and older. Regarding cardiac troponin, robust existing data show that increased values are predictive of all-cause mortality and amputations in septuagenarian PAD patients, as reported by Linnemann et al. [30, 31]. Indeed, increased cytokine and biomarker levels might reflect the overall ischemic burden, particularly that in patients with CLTI. Our observation that an increased baseline troponin level predicts mortality is corroborated by other studies showing a similar HR of 4.2 [32]. A striking and novel finding of the present study is that even continuous values of troponin are predictive of all-cause mortality in the elderly, while in younger patients, this relation was not evident. Of note, our predictive model applies mainly to patients who are undergoing endovascular interventions, as these were the patients included here.

Overall, the mortality rate in elderly patients with PAD remains undisputedly high. Etiological factors might include refraining from adherence to guideline‐recommended therapy, which has been shown to significantly reduce major adverse cardiovascular events (MACEs), major limb events (MALEs) and mortality [33, 34]. Despite the proven cardiovascular risks of PAD and established guidelines for the treatment of this widespread disease, studies continue to reveal that millions of PAD patients are undertreated [35–37]. Remarkably, our data show that octo- and nonagenarians seem to be treated even worse than younger patients regarding guideline-recommended therapies, such as antiplatelets and statins. Refraining from prescribing these medications might be due to misconceptions about possible adverse effects or drug interactions. We show that elderly patients are at an increased risk, which supports adherence to cardiovascular protective medication regimens. As shown recently, greater adherence to treatment with statins after cardiovascular events is associated with improved survival, which supports the concept of strict adherence to pharmacotherapy even in older patients [38].

Current guidelines further recommend that PAD patients cease smoking and receive antiplatelet therapy and treatment with statin medications and angiotensin‐converting enzyme (ACE) inhibitors for secondary prevention and cardiovascular risk reduction. We now show that elderly patients are undertreated as compared to younger ones, regarding essential medications like antiplatelets and statins. Our data provide further support for recent European Society of Cardiology (ESC) guidelines, which have implemented screening for cardiovascular comorbidities and recommend the determination of natriuretic peptides such as NT-proBNP [39]. With the current knowledge, in PAD, troponin and natriuretic peptide tests should be performed not only for screening CVD but also for predicting mortality.

Some limitations regarding our work must be considered. First and foremost, we could not present a matched cohort, which would be needed to compare elderly and younger individuals in depth regarding mortality. Furthermore, we did not perform risk factor adjudication due to the small sample size. In previous studies, the relative prognostic importance of traditional cardiovascular risk factors was determined, with tobacco use, a low ABI and diabetes being associated with an increased risk of mortality, while physical activity was associated with a decreased risk of mortality [40]. Thus, a potential confounding issue could exist, as these data were not available for our cohort. Nonetheless, as we used the database of all patients referred to our hospital, we believe that this fact can be neglected. Potential adaptation of the presented risk should be performed in larger studies with matched cohorts and detailed risk factor stratifications. Due to the explorative approach, we performed a monocentric retrospective study. Furthermore, our findings need to be proven predictive for nontertiary care center hospitals, as our results might be influenced by referral bias. Additionally, we did not include patients who were treated surgically based on the nature of our department (with a separate department for vascular surgery). However, we believe that endovascular techniques dominate the methods used to care for octo- and nonagenarians and especially frail people and that our results thus represent current treatment strategies. Clearly, further studies based on larger registries with longer follow-up periods could focus on detailed MACE or MALE data for optimal risk stratification and patient counseling.

For adequate patient counseling and optimal peri- and postinterventional management, a clear predictive algorithm is necessary. We provide novel data regarding risk stratification in octo- and nonagenarian patients, showing that the baseline troponin level in conjunction with the NT-proBNP level and Rutherford classification are the most important factors predicting mortality in this high-risk population. Importantly, continuous levels of troponin and NT proBNP were shown to be of predictive value and helpful in risk stratification. Determination of all factors should be vigorously implemented in routine clinical practice when treating octo- and nonagenarians and elderly patients with PAD.

## Supplementary Information

**Additional file 1**. Baseline laboratory characteristics, medications and procedural characteristic.

## Data Availability

The datasets used and/or analyzed during the current study are available from the corresponding author on reasonable request.

## Abbreviations

CLTI: Chronic limb-threatening ischemia
CVD: Cardiovascular disease
PAD: Peripheral artery disease
SD: Standard deviation
