# Supplementary material for: Risk stratification and mortality prediction in octo- and nonagenarians with peripheral artery disease: a retrospective analysis
Source: BMC Cardiovasc Disord. 2021 Aug 2;21:370. doi: 10.1186/s12872-021-02177-1 (PMC8330051; doi:10.1186/s12872-021-02177-1)
Supplement: Supplementary file 1 — Additional file 1. Baseline laboratory characteristics, medications and procedural characteristic. [file 12872_2021_2177_MOESM1_ESM.docx]

**Additional file 1**

**Risk stratification and mortality prediction in octo- and nonagenarians with peripheral artery disease**

Christos Rammos*, MD; Aristotelis Kontogiannis, MD; Amir A. Mahabadi, MD; Martin Steinmetz, MD; Daniel Messiha, MD; Julia Lortz, MD; MD; Tienush Rassaf, MD

### Additional file 1: Figure S1


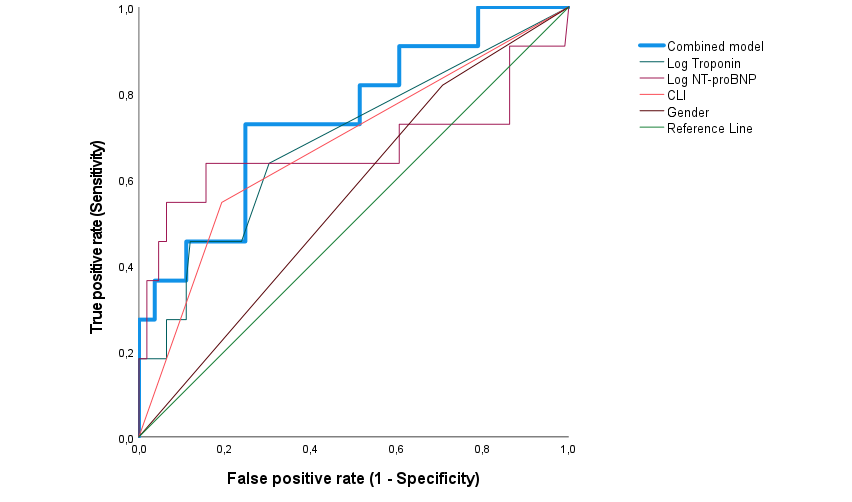


ROC curves for predicting all-cause mortality in sexa- and septuagenarians. ROC curve analysis showing the prognostic value of gender, CLI, log NT-proBNP, log troponin-ultra and a combination model including all factors.

### Additional file 1: Table S1. Clinical routine

|  | ≥ 80 years old  (n = 123) | < 80 years old  (n = 123) | P Value |
| --- | --- | --- | --- |
| Sodium, mmol/l | 139 ± 4 | 140 ± 3 | 0.27 |
| Potassium, mmol/l | 4.4 ± 0.5 | 4.5 ± 0.5 | 0.51 |
| eGFR, ml/min | 53 ± 18 | 66 ± 24 | 0.0001 |
| CK, U/l | 94 ± 113 | 99 ± 63 | 0.94 |
| Troponin I Ultra, ng/l | 528 ± 4500 | 32 ± 250 | 0.25 |
| NT-proBNP, pg/ml | 3120 ± 6158 | 4255 ± 29427 | 0.7 |
| AST(SGOT), U/l | 27 ± 27 | 22 ± 13 | 0.052 |
| ALT(SGPT), U/l | 26 ± 38 | 25 ± 19 | 0.85 |
| C reactive protein, mg/dl | 2.0 ± 4.2 | 0.9 ± 1.8 | 0.01 |
| Cholesterol, mg/dl | 186 ± 63 | 176 ± 43 | 0.215 |
| HDL, mg/dl | 56 ± 19 | 46 ± 16 | 0.0001 |
| LDL, mg/dl | 111 ± 44 | 107 ± 37 | 0.53 |
| Triglycerides | 138 ± 75 | 192 ± 127 | 0.0001 |

eGFR: estimated glomerular filtration rate, CK: Creatinine kinase, AST: Aspartat-Aminotransferase, ALT: Alanin- Aminotransferase. LDH: Lactate Dehydrogenase, HDL: High density lipoprotein, LDL: low density lipoprotein, NT-proBNP: N-terminal pro B-type natriuretic peptide, TSH: Thyroid stimulating hormone. Data are mean ± SD

### Additional file 1: Table S2. Procedural characteristics

|  | ≥ 80 years old  (n = 123) | < 80 years old  (n = 123) | P Value |
| --- | --- | --- | --- |
| Dose area product, μGy/m2 | 2648 (±5608) | 3020 (±4611) | 0.6 |
| Contrast agent. ml | 96 (±34) | 93 (±42) | 0.5 |
| Radiation time. min | 32 (±122) | 17 (±15) | 0.25 |
| Target lesion |  |  |  |
| Iliac | 16 (13%) | 40 (33%) | 0.0001 |
| Femoral | 90 (73%) | 79 (64%) |  |
| Crural | 17 (13%) | 4 (3%) |  |
| Type of lesion |  |  |  |
| Denovo | 113 (91%) | 107( 87%) | 0.221 |
| Restenosis | 10 (8%) | 16 (13%) |  |
| Occlusion | 63 (51%) | 54 (44%) | 0.250 |
| TASC |  |  |  |
| A | 6 (5%) | 23 (19%) | 0.0001 |
| B | 62 (50%) | 42 (34%) |  |
| C | 10 (8%) | 38 (31%) |  |
| D | 45 (35%) | 20 (16%) |  |
| PTA | 93 (75%) | 75 (61%) | 0.027 |
| DCB | 64 (52%) | 68 (55%) | 0.296 |
| Rotational Thrombectomy | 23 (19%) | 8 (7%) | 0.019 |
| Stent Implantation | 43 (35%) | 49 (40%) | 0.405 |
| Stent length | 97 (±73) | 62 (±72) | 0.015 |
| DCB length | 134 (±101) | 126 (±108) | 0.688 |

### Additional file 1: Table S3. Univariate analysis of mortality of patients with PAD

| Risk factors | ≥80 years old | | | <80 years old | | |
| --- | --- | --- | --- | --- | --- | --- |
|  | P value | HR | 95% CI | P value | HR | 95% CI |
| Troponin I, Ultra | <0.001 | 2.56 | 1.85-3.5 | 0.005 | 1.79 | 1.12-2.67 |
| NT-proBNP | <0.001 | 2.3 | 1.56-3.38 | 0.005 | 1.94 | 1.22-3.08 |
| CLTI | 0.005 | 3.07 | 1.4-6.8 | 0.001 | 6.22 | 2.02-19 |
| Gender | 0.46 | 1.29 | 0.66-2.51 | 0.61 | 0.68 | 0.15-3.1 |
| Diabetes mellitus | 0.27 | 1.2 | 0.86-1.7 | 0.771 | 0.92 | 0.51-1.64 |
| Smoking | 0.13 | 0.5 | 0.22-1.2 | 0.37 | 0.72 | 0.36-1.47 |
| Renal failure mild to severe | 0.37 | 1.39 | 0.68-2.83 | 0.06 | 2.2 | 0.95-8.9 |

CLTI: Chronic limb threating ischemia. HR: Hazard ratio, CI 95%: Confidence interval 95%
